# Snail coprophagy: the encounter filter, food preferences, and rat lungworm (*Angiostrongylus cantonensis*) prevalence[Fn FN1]

**DOI:** 10.1051/parasite/2024075

**Published:** 2024-12-23

**Authors:** Randi L. Rollins, Chasen D. Griffin, Robert H. Cowie

**Affiliations:** 1 Pacific Biosciences Research Center, University of Hawaii at Manoa Honolulu Hawaii USA; 2 School of Life Sciences, University of Hawaii at Manoa Honolulu Hawaii USA

**Keywords:** *Angiostrongylus cantonensis*, Combes filters, Gastropod, Parasite transmission, Rat feces, Zoonoses

## Abstract

Understanding the factors driving infection prevalence among host species is crucial for effective disease mitigation. *Angiostrongylus cantonensis*, the rat lungworm, causes neuroangiostrongyliasis and serves as an excellent model for studying infection dynamics across hosts. This study investigates the relative impact of encounter rates on *A. cantonensis* prevalence in snail hosts by assessing their coprophagic tendencies. Multiple-choice feeding assays were conducted with four snail species (*Parmarion martensi*, *Laevicaulis alte*, *Lissachatina fulica*, and *Veronicella cubensis*) differing in *A*. *cantonensis* prevalence. The snails were offered romaine lettuce, hibiscus flowers, papaya, and rat feces. The relative intake ratios (RIR) were calculated and used to evaluate 1) feces preference among the snail species, and 2) correlation between feces preference and *A. cantonensis* prevalence. We also compared preferences for feces from rats fed high-fat and balanced diets; no significant difference was observed. Feces made up the highest proportion of the diet of *P. martensi* (11.6%), followed by *V. cubensis* (7.8%), *L. fulica* (5.9%), and *L. alte* (5.1%). Additionally, *P. martensi* showed a significantly higher preference (RIR) than all other species. The correlation between feces preference and *A. cantonensis* prevalence among species was weakly positive. These findings suggest that the level of coprophagy influences encounter rates with *A. cantonensis*, contributing to variation in infection prevalence among snail species. However, other factors may also play a role, as preference and prevalence were only weakly correlated. Understanding these dynamics can inform strategies for managing the spread of *A. cantonensis* and mitigating its health impacts.

## Abstract (fr)

Comprendre les facteurs qui influencent la prévalence des infections chez les espèces hôtes est crucial pour une atténuation efficace des maladies. *Angiostrongylus cantonensis*, le ver pulmonaire du rat, provoque la neuroangiostrongylose et constitue un excellent modèle pour étudier les dynamiques d’infection à travers les hôtes. Cette étude examine l’impact relatif des taux de rencontre sur la prévalence de *A. cantonensis* chez les hôtes escargots en évaluant leurs tendances coprophagiques. Des essais d’alimentation à choix multiples ont été réalisés avec quatre espèces d’escargots (*Parmarion martensi*, *Laevicaulis alte*, *Lissachatina fulica* et *Veronicella cubensis*), différant par la prévalence d’*A. cantonensis*. De la laitue romaine, des fleurs d’hibiscus, de la papaye et des excréments de rats ont été proposés aux escargots. Les ratios d’ingestion relative (RIR) ont été calculés et utilisés pour évaluer 1) la préférence pour les excréments parmi les espèces d’escargots, et 2) la corrélation entre la préférence pour les excréments et la prévalence d’*A. cantonensis*. Nous avons également comparé les préférences pour les excréments de rats nourris avec des régimes riches en graisses et des régimes équilibrés, sans observer de différence significative. Les excréments représentaient la plus grande proportion du régime alimentaire pour *P. martensi* (11,6 %), suivi de *V. cubensis* (7,8 %), *L. fulica* (5,9 %) et *L. alte* (5,1 %). De plus, *P. martensi* a montré une préférence (RIR) significativement plus élevée que toutes les autres espèces. La corrélation entre la préférence pour les excréments et la prévalence de *A. cantonensis* parmi les espèces était faiblement positive. Ces résultats suggèrent que le niveau de coprophagie influence les taux de rencontre avec *A. cantonensis*, contribuant à la variation de la prévalence des infections chez les espèces d’escargots. Cependant, d’autres facteurs jouent également un rôle, comme l’indique la faible corrélation entre la préférence pour les excréments et la prévalence des infections. Comprendre ces dynamiques peut informer les stratégies de gestion de la propagation de *A. cantonensis* et atténuer son impact sur la santé.

## Introduction

Variation in infection prevalence and intensity among host species is a common phenomenon in multi-host-parasite systems [51, 66, 73]. Understanding the drivers of this variation in zoonotic parasite systems is a crucial component of developing effective disease mitigation strategies [8, 62, 72]. The Combes filter concept [13, 23] provides a framework to understand the mechanisms underlying heterogeneous infection patterns among host species. It proposes that host encounter rate and host compatibility drive these variations. The “encounter filter” addresses the potential for a host to encounter a parasite at the appropriate stage in its life-cycle; the “compatibility filter” addresses the parasite’s ability to establish and develop within a given host. Despite the importance of both filters in determining host suitability, it remains challenging to measure their relative contributions in natural host-parasite transmission cycles [41]. While compatibility can be evaluated by experimental infection and observation of parasite development in a laboratory setting, assessing encounter rates is more difficult.

*Angiostrongylus cantonensis* (Chen, 1935) [12], commonly known as the rat lungworm, is a tropical/subtropical parasitic nematode with a complex life-cycle requiring rats as definitive hosts and snails/slugs (hereafter “snails”) as intermediate hosts [16]. This parasite system serves as an excellent model for investigating infection dynamics across hosts because many snail species across the gastropod phylogeny serve as intermediate hosts for the parasite [36, 37, 65]*,* and substantial variation in *A. cantonensis* prevalence and intensity exists among snail species [37, 57], even among closely related species residing in the same area [45, 57]. Moreover, *A. cantonensis* is the causative agent of neuroangiostrongyliasis, an emerging but neglected tropical disease and a leading cause of human eosinophilic meningitis globally [4, 16, 70]. The parasite can also cause severe neurological disease in other vertebrates, including dogs, horses, primates, marsupials, and birds [15]. Therefore, investigating the drivers of infection patterns among host species could improve prevention of human and animal infection.

*Angiostrongylus cantonensis* is acquired by snails primarily through ingestion of infected rat feces. First-stage larvae are expelled in rat feces and ingested by snails, in which they develop into third-stage larvae. Infected snails are then consumed by rats, in which the larvae mature and reproduce. Once the eggs hatch and the rats excrete first-stage larvae in their feces, the cycle continues (*e.g.* [70]). Therefore, the coprophagic tendencies of a snail species can be considered an indirect measure of its encounter rate with *A. cantonensis*. By comparing the relative preferences of different snail species for rat feces with their known infection prevalences, we may be able to gain understanding of the respective roles of the encounter and compatibility filters, and how they shape infection patterns across host species. For example, if species exhibiting high infection prevalence strongly prefer rat feces, while those with low prevalence do not, heterogeneous encounter rates among species may be inferred. Conversely, if preferences for rat feces among snail species do not align with their infection prevalences, compatibility rather than encounter may be driving the variation in prevalence.

By focusing on a model system with clear ecological and epidemiological relevance, we set out to identify general principles governing infectious disease dynamics in wildlife populations. Specifically, this study aimed to determine the relative impact of the Combes filters on *A. cantonensis* prevalence in snails by assessing food preferences, including coprophagic tendencies, in different species. We hypothesized that 1) snail species exhibit differing food preferences, particularly for rat feces, and 2) species with high *A. cantonensis* prevalences have a stronger preference for rat feces than species with low prevalences. To test these hypotheses, we conducted multiple-choice feeding assays with four snail species, each exhibiting different levels of *A. cantonensis* prevalence. By examining the feeding preferences of these species, we aimed to elucidate the role of host encounter rate in shaping *A. cantonensis* infection patterns among intermediate hosts.

## Materials and methods

### Snails and food types

In addition to rat feces, the following three food types were selected for the assays because of their known roles in the diets of our study species: romaine lettuce, hibiscus flowers, and papaya ([29, 71], Rollins unpublished]. Lettuce and papaya were purchased from a local grocery store, while mature hibiscus flowers were collected from the University of Hawaiʻi at Manoa campus. Rat fecal pellets were sourced from two groups of *Rattus norvegicus*: one fed a high-fat diet and another fed a balanced diet (Oxbow Essentials Adult Rat Food supplemented with nuts and fruits), which allowed us to evaluate the effect of feces composition on snail coprophagy. The high-fat diet pellets were acquired from laboratory rats housed at the University of Hawaiʻi and the balanced diet pellets came from privately owned rats purchased from a local pet store. Rats were treated humanely and in accordance with the University of Hawaiʻi’s Animal Welfare Program, following approval from the Institutional Animal Care and Use Committee (IACUC) under protocol #21-3499-4 and the Institutional Biosafety Committee (IBC) under approval #B24-101182.

We tested the food preferences of 192 snails, with 48 individuals representing each of the following four species: *Parmarion martensi* (Ariophantidae), *Laevicaulis alte* (Veronicellidae), *Lissachatina fulica* (Achatinidae), and *Veronicella cubensis* (Veronicellidae). Reproductively mature individuals, determined by size and/or weight ([27, 67], Rollins unpublished), were collected from wild populations in Manoa Valley on the island of Oʻahu, Hawaiʻi (where they are invasive) and brought to the laboratory. They were housed in plastic containers for 24–48 h without food, before beginning the feeding assay so that they were willing to feed. Containers were misted to prevent dehydration and kept at 23 °C under a 12-h light/dark cycle.

### Feeding arena and assay

**Figure 1:**
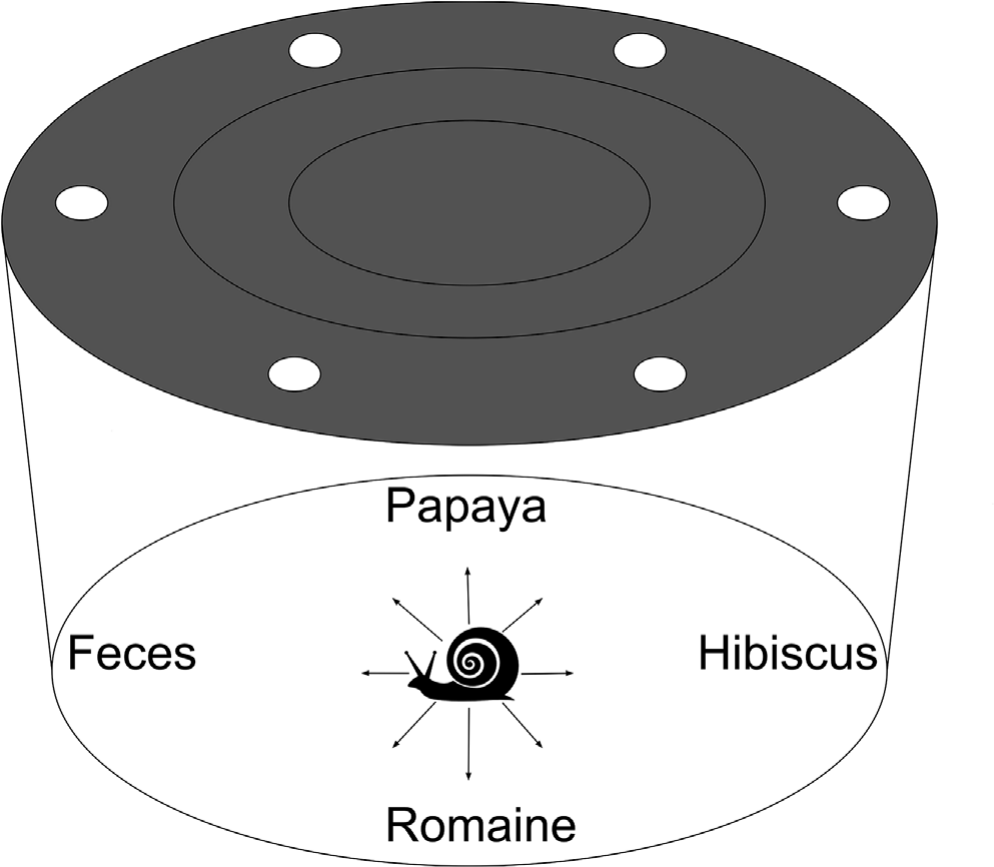
Diagram of circular 27.5 cm × 14 cm food choice arena. Snails were oriented randomly at the beginning of each food choice trial.

Twenty-four food choice arenas were constructed using round (27.5 cm diameter, 14 cm height) clear plastic cake domes, each inverted and with six ventilation holes. The four food types were placed equal distances apart at the periphery of each arena, and a single snail was positioned in the center (Fig. 1).

The food choice trials ran for 24 h. Each trial consisted of 24 snails (one in each arena), six of each species. Eight trials were conducted, thus testing 192 snails. The trials were identical, except that the first four trials used feces from rats fed a high-fat diet, while the second four trials used feces from rats fed a balanced diet (see above). Foods were weighed and placed at the periphery of the arenas in the following order, clockwise: papaya, hibiscus, lettuce, rat feces. Snails were also weighed at the start of the trial and their initial orientation in the center of the arena was randomized. Arenas were monitored hourly and foods replenished as needed to ensure a constant supply, avoiding a ceiling effect. After the 24 h trial period, the remaining food was weighed.

Data from 8 snails were excluded from the analysis because of snail excretion on the food, which interfered with accurate weights, leaving data from 184 snails (44 *L. fulica*, 46 *P. martensi*, 47 *L. alte*, and 47 *V. cubensis*) for downstream analysis.

### Data

Food intake was calculated for each snail as the difference between start and end weight for each food type, measured to 0.01 mg. To account for variations in the amount of food offered and size differences among the species, the food intake data were standardized by transforming them into a relative intake ratio (RIR), calculated as:

RIR = (mg food type X ingested / mg all food ingested) / (mg food X offered / mg all food offered).

This standardization allowed us to compare the proportion of each food type ingested to the proportion offered among species. A higher RIR indicates a stronger preference, while a lower RIR indicates a lower preference for the food type.

### Statistical analysis

All statistical analyses were performed using R Statistical Software (v4.3.0) [53]. An independent *t*-test was used to compare the RIRs of high-fat diet feces with balanced diet feces. Separate Kruskal–Wallis tests were conducted for each of the four food types (papaya, lettuce, hibiscus, and feces) to assess differences in food preferences among the four snail species. Given the primary interest in feces preference, pairwise comparisons between the snail species were carried out using a *post hoc* Dunn’s test to identify specific differences in RIR for feces, with the dunn.test R package [20]. To control for multiple comparisons, *p*-values were adjusted using the Bonferroni correction method, with the significance level adjusted accordingly based on the number of comparisons. The significance level for all tests was set at *α* = 0.05. To evaluate the relationship between feces RIR and *A. cantonensis* prevalence among the species, we searched published literature for *A. cantonensis* prevalence data for each of the four snail species (Table 1). From these data, we calculated the median prevalence for each species and used it to obtain a Spearman’s rank correlation coefficient of feces RIR and *A. cantonensis* prevalence.

**Table 1:** *Angiostrongylus cantonensis* prevalence (% of individuals infected) and sample size (*n*) in snail species in representative published literature.

Location	*L. alte* (n)	*L. fulica* (n)	*P. martensi* (n)	*V. cubensis* (n)	Source
Thailand		4.8 (1,017)			Crook et al*.* 1968 [17]
New Caledonia	28.8 (545)				Ash 1976 [3]
Indonesia		23.0 (283)			Carney et al*.* 1978 [10]
Papua New Guinea		31.0 (200)			Scrimgeour & Welch 1984 [59]
India	44.0 (75)				Limaye et al*.* 1988 [43]
Okinawa (Japan)		26.1 (4,872)	20.3 (753)		Asato et al*.* 2004 [2]
Ogasawara (Japan)		32.7 (449)			Suzuki et al*.* 2004 [63]
Hawaii (USA)			77.5 (40)		Hollingsworth et al*.* 2007 [29]
Brazil		36.8 (15)			Thiengo et al*.* 2010 [64]
China		25.0 (3,184)			Chen et al*.* 2011 [11]
Thailand		12.4 (307)			Vitta et al*.* 2011 [69]
China		14.0 (795)			Yang et al*.* 2012 [74]
Philippines		16.7 (365)			Constantino-Santos et al*.* 2014 [14]
Hawaii (USA)	29.5 (44)	11.3 (62)	68.4 (19)	2.5 (159)	Kim et al*.* 2014 [37]
Florida (USA)		36.0 (50)			Iwanowicz et al*.* 2015 [34]
Florida (USA)		23.9 (592)			Smith et al*.* 2015 [60]
Guadeloupe		32.4 (34)			Dard et al*.* 2017 [18]
China		10.3 (302)			Huang et al*.* 2017 [32]
Brazil		18.5 (110)			Ramos-de-Souza et al*.* 2018 [55]
Hawaii (USA)	38.4 (52)	28.3 (177)	63.8 (29)	2.8 (150)	Medeiros et al*.* 2020 / Rollins et al*.* 2021 (same data) [45, 57]
Hawaii (USA)			86.2 (159)		Niebuhr et al*.* 2021 [48]
Brazil		7.1 (44)			Ramos-de-Souza et al*.* 2023 [54]
Hawaii (USA)			75.3 (196)		Rollins et al*.* 2023 [56]
China		47.1 (187)			Zhang et al*.* 2023 [75]
Median prevalence	34.0 (716)	23.9 (13,045)	71.9 (1,196)	2.7 (309)	

## Results

### High-fat *vs.* balanced diet rat feces

No significant difference was observed in the preference for feces (RIR for feces) from high-fat diet rats ($\bar{x}=0.777$, SD = 0.383) and balanced diet rats ($\bar{x}\;=\;0.776$, SD = 0.396) (*t*(182) = 0.026, *p* = 0.979). Therefore, the data from both feces types were combined for the analyses.

### Composition of diet preferences

**Figure 2:**
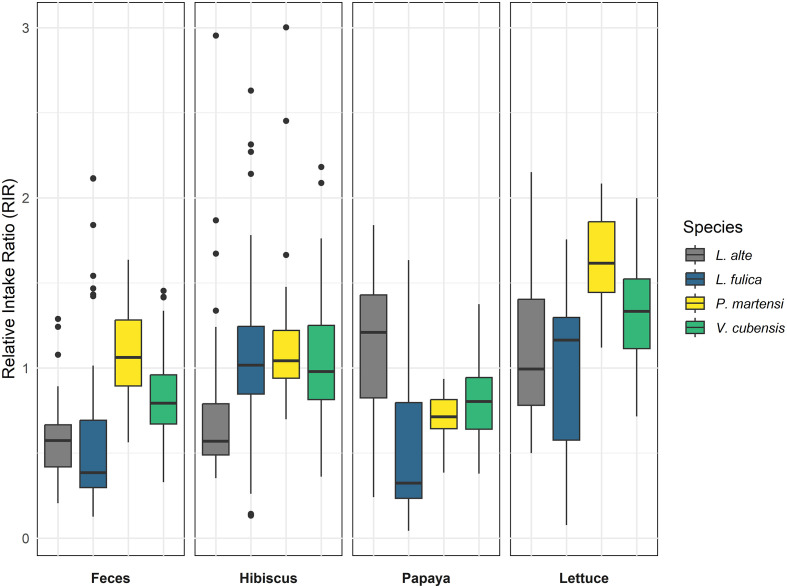
The relative intake ratio (RIR) (*y*-axis) represents food preference for each snail species (*x*-axis), for each food type. Boxplots represent data from 47 *L. alte*, 44 *L. fulica*, 46 *P. martensi*, and 47 *V. cubensis*. The box represents the interquartile range (IQR) with the median indicated by the central line. Whiskers extend 1.5 times the interquartile range, and data points outside this range are plotted as outliers.

The RIR and proportion of each food type per species highlight the varied food preferences among the four species (Table 2; Fig. 2). *Parmarion martensi* showed a relatively balanced preference between lettuce and papaya, with a higher proportion of rat feces consumption than any other species. *Veronicella cubensis* preferred lettuce over all other foods, with papaya as a close second. Meanwhile, *Lissachatina fulica* exhibited a marked preference for lettuce and had the highest proportion of hibiscus consumption among the species. *Laevicaulis alte* preferred papaya and lettuce, consuming the lowest proportion of feces compared to the other snails.

**Table 2:** Snail diet composition from the food choice trials. Percentage of each food type making up the snails’ diet (top), and its associated RIR (bottom), averaged by species.

Proportion of the diet				
Species	Papaya	Lettuce	Hibiscus	Rat feces
*L. alte*	43.5%	39.5%	11.9%	5.1%
*L. fulica*	17.0%	51.9%	25.2%	5.9%
*P. martensi*	38.3%	35.8%	14.3%	11.6%
*V. cubensis*	37.6%	40.7%	14.0%	7.8%
Relative intake ratio (RIR)				
Species	Papaya	Lettuce	Hibiscus	Rat feces
*L. alte*	1.09	1.10	0.74	0.59
*L. fulica*	0.55	1.00	1.07	0.61
*P. martensi*	0.72	1.61	1.15	1.08
*V. cubensis*	0.80	1.33	1.04	0.84

### Rat feces preferences

Rat feces constituted the highest proportion of the diet of *P. martensi* (11.6%), followed by those of *V. cubensis* (7.8%), *L. fulica* (5.9%), and *L. alte* (5.1%) (Table 2). This indicates that *P. martensi* had a stronger preference for feces as a food source than did the other three species (Fig. 2). These preference differences were statistically supported. The Kruskal–Wallis tests showed significant differences in the RIRs of all food types among the four snail species, including the RIR of rat feces (Table 3). Pairwise comparisons of feces RIR using the Dunn’s test indicated that the preference of *P. martensi* for rat feces differed significantly from those of each of the other three species (Table 4). The preference of *V. cubensis* also differed significantly from those of *L. alte* and *L. fulica*, while those of these latter two species did not differ significantly from each other (Table 4).

**Table 3:** Results of the Kruskal–Wallis tests comparing the relative intake ratio (RIR) among different snail species for each of four food types. The table displays the test statistic (χ^2^), degrees of freedom (df) and *p*-value for each food type. Significant values (*p* < 0.05) indicate that snail species differ in their preference for the given food type.

Food type	χ^2^	Df	*p*-value
Feces	66.9	3	1.95E–14*
Hibiscus	32.7	3	3.81E–07*
Lettuce	55.7	3	4.97E–12*
Papaya	42.7	3	2.82E–09*

Significance (*p* < 0.05) denoted by *.

**Table 4:** Results of Dunn’s *post hoc* test for pairwise comparisons of the relative intake ratio (RIR) for rat feces among different snail species. The table displays the *Z* values (test statistics) and the adjusted *p*-values (using Bonferroni correction) for each pairwise comparison. The *Z* value indicates the strength and direction of the difference between species pairs. If *Z* > 0, the first species in the comparison has a greater RIR than the second species. If *Z* < 0, the second species in the comparison has a greater RIR than the first species. Significant *p*-values (*p* < 0.05) are asterisked and indicate that preference for rat feces for the corresponding species pair differed significantly.

Comparison	*Z*	*p*-adjusted
*L. alte – L. fulica*	0.75	1.00E+00
*L. alte – P. martensi*	−6.49	5.07E−10*
*L. fulica – P. martensi*	−7.16	4.69E−12*
*L. alte – V. cubensis*	−3.65	1.58E−03*
*L. fulica – V. cubensis*	−4.36	7.87E−05*
*P. martensi – V. cubensis*	2.88	2.36E−02*

### Feces preference and *A. cantonensis* infection prevalence

Our findings on snail food preferences partly align with previously published *A. cantonensis* prevalence values of these four snail species from around the world (Table 1), which varied somewhat among these studies (which is not surprising considering the studies sampled from different locations and at different times of year). *Parmarion martensi* showed the strongest preference for rat feces compared to the other species, consistent with it having the highest median reported prevalence among the studies reviewed. Similar feces preferences and *A. cantonensis* prevalences were also exhibited by *L. alte* and *L. fulica*, with no significant differences in feces preferences in our study and median prevalences of 34.0% and 23.9%, respectively. However, the higher preference for rat feces of *V. cubensis* did not align with its comparatively lower median prevalence. The Spearman’s rank correlation was statistically significant and indicated a weak positive correlation between species’ preference for rat feces (RIR) and *A. cantonensis* prevalence, *r*(2) = 0.209, *p* = 0.005).

## Discussion

The results of this study highlight distinct dietary preferences among the four snail species tested, specifically for rat feces as a food source. These preferences therefore suggest that encounter rates with *A*. *cantonensis* larvae differ among snail species and may be involved in part in driving variation in their infection prevalences. For example, the pronounced preference of *P. martensi* for rat feces (Table 2; Fig. 2), which exhibits the highest median infection prevalence in the wild (Table 1), supports the hypothesis that host encounter rate, mediated through coprophagy, may be a component of *A. cantonensis* transmission in some host species, and highlights the ecological and epidemiological relevance of host encounter rates in parasite transmission dynamics.

The diverse dietary strategies of snails, ranging from polyphagous to highly specialized [7, 49], and spanning herbivory, detritivory, and carnivory [9, 61], underscore a complex ecological web in which food availability and nutritional needs may drive behavioral adaptations [1]. Coprophagy, while less common, is a behavioral adaptation that some species use to maximize nutrient intake, particularly in nutrient-poor environments [24, 26, 39]. It is also an example of how environmental and biological factors converge in the life-cycles of parasites. For instance, snails that consume rat feces inadvertently acquire *A. cantonensis* larvae, a crucial step in this parasite’s life-cycle. This mode of transmission, while straightforward, is likely to be nuanced by the variability in how different snail species interact with their environments. The marked preference for rat feces exhibited by *Parmarion martensi* may explain, at least partly, this species’ generally high infection rates in natural populations (Table 1), as increased coprophagy enhances exposure to *A. cantonensis* larvae.

The ecological implications of coprophagy extend beyond mere nutrient acquisition. Coprophagy can modulate gut microbial diversity and function [6, 22, 35, 47], which facilitates digestive processes and can influence host health and nutrition [30, 33, 50, 58], as well as snail behavior and physiology [19]. Such microbial-mediated effects can further interact with host susceptibility to parasites, as observed with *Plasmodium berghei* (malaria) in *Anopheles* mosquitos [44] and mice [68]. These interactions between diet, microbiome, and host susceptibility to parasites add another layer of complexity to infection dynamics [5, 28]. However, the role of the microbiome in parasite-transmitting snails is but a nascent area of research. While there have been studies exploring the snail microbiome [31, 42], direct investigations into how these microbial communities impact parasite transmission dynamics remain limited.

The feeding assays conducted in this study revealed distinct dietary preferences, with significant variability among species in their consumption of rat feces. While *P. martensi* showed the highest preference for feces and the highest infection prevalence, the lower preferences of *L. alte* and *L. fulica* also correlated with their relatively lower *A. cantonensis* infection prevalences. This aligns with findings from other studies, such as that of Garvon & Bird (2005) [25], which showed that intermediate snail hosts of *Parelaphostrongylus tenuis* (a deer brain worm) are attracted to deer excrement, which contains the parasite larvae, potentially increasing their risk of infection. Likewise, Křivan (1997) [40] observed that patterns of host feeding by parasitoids critically influence host-parasitoid population dynamics, in which different feeding strategies can either stabilize or destabilize these interactions depending on the ecological context. And, it has long been known that nonrandom host choice by mosquitos significantly affects the dynamics of malarial transmission, potentially altering infection rates and stability within host populations [38]. Such behaviors suggest that encounter rates, as mediated through dietary choices, significantly impact transmission dynamics.

The differences in dietary preferences among closely related and cohabitating species, such as *V. cubensis* and *L. alte*, further emphasize the complexity of host-parasite relationships. Despite being in the same family (Veronicellidae), and often found micro-sympatrically in the wild, their infection prevalences differ greatly (Table 3), even among cohabitating populations [45, 57]. Our study found that these two species exhibited different preferences for rat feces, which did not directly correspond to their *A. cantonensis* infection prevalences. The weak, though significant, correlation between feces preference and parasite prevalence in our study (perhaps due in some part to our testing only four species), calls for a deeper examination of other influencing factors such as host compatibility and immune defenses. Kuris et al. (2007) [41] highlighted the challenge in quantifying the distinct roles of encounter and compatibility in natural systems, pointing out the complexity when related hosts share overlapping habitats. This underscores that even closely related species, and species inhabiting the same microgeographical space, can have significantly different behaviors, ecological interactions, and parasite compatibility that influence their roles as hosts in parasite transmission cycles.

We would be remiss to ignore the evolutionary aspects of dietary preferences in snails, which have adapted to a range of food sources, as noted above. The co-evolutionary history of *P. martensi* and *A. cantonensis*, possibly sharing a native range in southern China and Southeast Asia [15, 21, 29, 52], may have shaped the strong coprophagic preference and susceptibility to *A. cantonensis* infection of *P. martensi*. For example, co-evolutionary feedback loops, as seen between *Biomphalaria* spp. and *Schistosoma mansoni*, in which the presence of *S. mansoni* exerts selective pressure on the snail populations, favoring traits that enhance compatibility with the parasite [46], may have refined these preferences, aligning snail behavior with *A. cantonensis* transmission dynamics. This complexity suggests that while behavioral traits like coprophagy are vital in parasite transmission, they are part of a broader ecological and evolutionary context that influences infection dynamics.

Further research is needed to fully determine the mechanisms underlying the variable *A*. *cantonensis* infection rates observed among snail species. The findings from this study provide a platform for future research, particularly in exploring the genetic and immunological factors that may influence the susceptibility and resistance of snails to *A. cantonensis*, and addressing the encounter filter’s companion, the compatibility filter. Exploring the impact of environmental variables, such as food availability and habitat preferences, on coprophagic behavior and infection prevalence could further clarify the ecological drivers of parasite transmission. Understanding these aspects could offer new insights into controlling the spread of *A. cantonensis*, with implications for broader ecological health and disease management strategies.

## Conclusion

While coprophagy in snail species acts as a bridge in the life-cycle of *A. cantonensis*, it also exemplifies the balance between behavior, ecology, and evolution in shaping disease transmission patterns. This study adds depth to our understanding of parasitic infections and highlights the complex interplay of biological and environmental factors that govern these dynamics. By illuminating the role of host encounter rates and dietary preferences in parasite transmission, we can better predict and manage the spread of zoonotic diseases, ultimately improving both human and domestic animal health.

## Data Availability

R code and xlsx files containing data can be found here: https://github.com/randirollins/encounter-filter.
